# Gleason Pattern 4 Burden and Adverse Pathology in ISUP Grade Group 2 Prostate Cancer: Implications for Active Surveillance Selection

**DOI:** 10.3390/cancers18193222

**Published:** 2026-10-06

**Authors:** Lucas Regis, Clara Perez, Jacques Planas, Ana Celma, Merce Cuadras, Maria-Eugenia Semidey, Richard Mast, Juan Morote, Enrique Trilla

**Affiliations:** 1 Department of Urology, Vall d’Hebron University Hospital, Vall d’Hebron Research Institute (VHIR), 08025 Barcelona, Spain; clara.perez@vallhebron.cat (C.P.); jacques.planas@vallhebron.cat (J.P.); ana.celma@vallhebron.cat (A.C.); merce.cuadras@vallhebron.cat (M.C.); enrique.trilla@vallhebron.cat (E.T.); 2 Department of Pathology, Vall d’Hebron University Hospital, 08025 Barcelona, Spain; maru.semidey@vallhebron.cat; 3 Department of Radiology, Institut de Diagnòstic per la Imatge (IDI), Hospital Universitari Vall d’Hebron, 08025 Barcelona, Spain; richard.mast@vallhebron.cat; 4 Universitat Autònoma de Barcelona, 08193 Barcelona, Spain; juan.morote@uab.cat

**Keywords:** prostate cancer, active surveillance, Gleason pattern 4, PSA density, adverse pathology, robot-assisted radical prostatectomy

## Abstract

Background: Active surveillance (AS) is increasingly considered for selected patients with International Society of Urological Pathology (ISUP) Grade Group (GG) 2 prostate cancer. However, candidate selection remains controversial due to histological heterogeneity and the risk of harboring adverse pathology (AP). We aimed to identify preoperative clinical, multiparametric magnetic resonance imaging (mpMRI), and histological predictors of AP, biochemical recurrence (BCR), and postoperative PSMA PET/CT positivity in men with biopsy-confirmed ISUP GG 2 disease undergoing robotic-assisted radical prostatectomy (RARP). Methods: We retrospectively analyzed 127 consecutive patients with biopsy-proven ISUP GG 2 prostate adenocarcinoma from a prospective single-center RARP database (2012–2024). All patients underwent pre-biopsy 3T mpMRI and systematic plus software-fusion targeted biopsies, with diagnostic specimens strictly lacking cribriform architecture or intraductal carcinoma. AP was defined as pathological stage ≥ pT3 and/or ISUP GG upgrading to ≥3 in the final surgical specimen. BCR was defined as two consecutive postoperative PSA measurements ≥ 0.2 ng/mL. Patients with BCR underwent PSMA PET/CT restaging. Univariable and multivariable logistic regression analyses evaluated independent preoperative predictors of AP, incorporating separate models for ≥10% and ≥15 biopsy Gleason pattern 4 thresholds to prevent collinearity. Model performance was evaluated using the area under the receiver operating characteristic curve (AUC) and Hosmer–Lemeshow calibration tests. Results: Median patient age was 63.2 years, median PSA was 7.6 ng/mL, and median PSA density (PSAD) was 0.18 ng/mL. PI-RADS ≥ 4 lesions were identified in 71.6% of patients, and median biopsy pattern 4 proportion was 21.1%. Overall, AP occurred in 39 of 127 patients (30.7%), driven by ISUP upgrading to GG ≥ 3 in 21.3% (n = 27) and upstaging to ≥pT3 in 17.3% (n = 22). No undergrading was observed. On univariable analysis, PSA (*p* = 0.028), PSAD (*p* = 0.011), and biopsy pattern 4 proportion ≥ 10% (*p* = 0.042) and ≥15% (*p* = 0.027) were significantly associated with AP. On multivariable analysis, PSAD (OR 6.75, 95% CI 3.51–12.87, *p* = 0.005) and biopsy pattern 4 proportion ≥ 10% (OR 2.54, 95% CI 1.15–6.54, *p* = 0.036) or ≥15% (OR 2.74, 95% CI 1.06–6.06, *p* = 0.023) remained independent predictors of AP. The final multivariable model demonstrated solid discrimination (AUC = 0.78, 95% CI 0.71–0.85) and satisfactory calibration (*p* = 0.42). At a mean follow-up of 32.3 months, BCR occurred in 23 patients (18.1%; 3-year recurrence-free survival: 81.3%). Restaging PSMA PET/CT was positive in 8 of 23 recurrences (34.8%; three local, four regional nodal, one solitary bone). Neither clinical nor radiological parameters independently predicted BCR or PSMA PET/CT positivity. Conclusions: Biopsy Gleason pattern 4 extent (<10% and <15%) and PSAD are the strongest independent preoperative predictors of organ-confined, non-upgraded pathology in ISUP GG 2 prostate cancer. Combining a low pattern 4 burden (<10%, or up to 15% in highly selected cases) with a low PSAD provides objective parameters to refine “favorable intermediate-risk” stratification by identifying patients with a lower probability of adverse pathological features.

## Simple Summary

Selecting men with ISUP Grade Group (GG) 2 prostate cancer for active surveillance remains challenging. In this Flow, 10% (and up to 15% in selected cases) combined with low PSA density reliably identifies organ-confined, non-upgraded pathology. These quantitative thresholds provide objective criteria to refine surveillance eligibility and prevent overtreatment.

## 1. Introduction

The widespread use of prostate-specific antigen (PSA) testing has shifted diagnosis toward localized and lower-risk disease [1,2]. While this stage migration allows curative treatment for many, it also raises concerns about overtreatment and unnecessary morbidity in men with indolent tumors [3,4].

Active surveillance (AS) has become the preferred management strategy for most patients with low-risk disease, providing excellent long-term cancer-specific survival and preservation of quality of life [3,4,5]. However, the role of AS in intermediate-risk PCa, particularly International Society of Urological Pathology (ISUP) Grade Group (GG) 2 (Gleason score 3+4), remains debated [6,7]. These tumors are biologically heterogeneous: while many display indolent behavior similar to GG1 cancers, others harbor aggressive features evident only after surgery [8,9].

Identifying the subset of ISUP GG 2 prostate cancers with indolent biological behavior is essential to safely expand active surveillance (AS) beyond Grade Group 1 disease. Accumulating data suggests that not all ISUP GG 2 tumors behave uniformly; many exhibit outcomes comparable to low-risk cancers, particularly when the proportion of Gleason pattern 4 is minimal [10]. The percentage of pattern 4 in biopsy cores has emerged as a strong pathological indicator of aggressiveness, correlating with adverse pathology, biochemical recurrence, and metastasis-free survival after radical prostatectomy [10,11,12]. Patients with low pattern 4 demonstrate similar oncological outcomes to ISUP 1 disease, supporting the safety of deferred intervention in carefully selected cases [11,12]. This refinement could prevent overtreatment while preserving the window for cure in men with favorable ISUP GG 2 PCa.

Contemporary studies demonstrate the value of integrating clinical and imaging parameters to predict adverse pathology at radical prostatectomy (RP) in men with ISUP GG 2 PCa [13,14]. Multiparametric MRI (mpMRI) further refines risk stratification, as higher PI-RADS scores, larger lesion size, and capsular contact length independently predict adverse pathology [13,14]. Furthermore, primary PSMA PET/CT imaging has demonstrated superior accuracy over conventional modalities for staging prostate cancer, offering enhanced cost-effectiveness and targeted detection of occult systemic disease [15,16]. The combination of these modalities enables a more individualized assessment of tumor aggressiveness before robot-assisted laparoscopic prostatectomy (RARP).

Despite these insights, there remains limited understanding of which preoperative factors—clinical, imaging, or pathological—best predict adverse final pathology, biochemical recurrence (BCR), or postoperative PSMA PET/CT positivity among men with ISUP GG 2 disease undergoing surgery.

We therefore aimed to identify preoperative clinical, imaging, and pathological predictors of adverse final pathology, biochemical recurrence, and positive postoperative PSMA PET findings in patients with ISUP GG 2 prostate cancer treated with RARP, to refine risk stratification and guide individualized management strategies.

## 2. Material and Methods

We conducted a retrospective analysis of a prospectively maintained institutional database including 907 consecutive patients who underwent RARP between 2012 and 2024. Among them, 127 patients had biopsy-confirmed ISUP GG 2 PCa and complete preoperative, pathological, and follow-up data available for analysis. Preoperative variables collected included age, prostate-specific antigen (PSA), PSA density (PSAD), multiparametric MRI findings (PI-RADS score and clinical stage), number of cancerous cores on biopsy, and pattern 4 proportion of the biopsy sample. Radiological staging was assessed according to ESUR PI-RADS v2 criteria, and clinical staging was defined using the 2017 TNM classification.

### Biopsy and MRI Protocols

All patients underwent systematic transrectal or transperineal prostate biopsy according to institutional protocols and contemporary European Association of Urology (EAU) guidelines. A minimum of 12 systematic cores were obtained under ultrasound guidance, targeting bilateral apex, mid-gland, and base regions. In cases with mpMRI-detected lesions, additional targeted biopsies (2–4 cores per lesion) were performed using MRI/ultrasound software-fusion guidance. Biopsies were performed under MRI-TRUS fusion guidance using either the BK3000 system (BK Medical) or the KOELIS Trinity® platform with Organ-Based Tracking (KOELIS). 

Histological assessment was performed by dedicated subspecialized uropathologists at a single tertiary academic center. Although our institutional RARP registry spans from 2012 to 2024 (n = 907), the final study sub-cohort of 127 consecutive patients with biopsy-confirmed ISUP Grade Group 2 disease and complete clinical, pathological, and follow-up data was accrued between 2016 and 2024.

All biopsy and radical prostatectomy specimens underwent centralized histological review according to the ISUP grading standards. To ensure complete diagnostic uniformity across the entire inclusion period, historical biopsy specimens were retrospectively re-evaluated by dedicated uropathologists according to updated ISUP/GUPS consensus guidelines. Specifically, for earlier specimens where granular pattern 4 percentages or detailed architectural subtype descriptions were not explicitly detailed in the original narrative report, slides were retrospectively re-examined to quantify pattern 4 extent and systematically confirm the presence or absence of cribriform pattern 4 and intraductal carcinoma.

To accurately reflect individual core involvement, the pattern 4 burden was operationalized as the highest percentage (maximal rate) of pattern 4 reported in any single biopsy core/container for each patient. The total number of positive cores, maximum cancer length per core, and overall cancer involvement were systematically recorded.

All patients underwent mpMRI prior to biopsy on a 1.5-Tesla or 3.0-Tesla scanner equipped with a phased-array surface coil, following institutional multiparametric protocols including high-resolution T2-weighted imaging in three planes, diffusion-weighted imaging (b-values up to 1400 s/mm^2^), and dynamic contrast-enhanced sequences. Images were interpreted by dedicated abdominal radiologists with at least 10 years of experience in prostate imaging, blinded to clinical and histopathological data.

Suspicious lesions were categorized based on contemporaneous institutional reports applying the PI-RADS v2.0 (for scans performed between May 2016 and 2019) or PI-RADS v2.1 system (from 2019 onward). The highest-scoring lesion per patient was selected for primary analysis. Extraprostatic extension, seminal vesicle invasion, and clinical stage (cT category) were recorded. MRI findings were subsequently correlated with final radical prostatectomy outcomes to evaluate local staging accuracy.

Final specimen pathological evaluation followed ISUP 2014 grading recommendations. Adverse pathology (AP) was defined as ≥pT3 disease and/or upgrading to ISUP GG ≥ 3 in the final specimen. Biochemical recurrence (BCR) was defined as a postoperative PSA ≥0.2 ng/mL confirmed by a second measurement. In patients experiencing BCR, PSMA PET/CT imaging was performed using 18F-PSMA-1007 or 68Ga-PSMA-11. PSMA PET/CT findings were reviewed when available. Univariable and multivariable logistic regression analyses were performed to identify predictors of AP, BCR, and positive PSMA PET/CT imaging. Statistical analyses were carried out using IBM SPSS Statistics for Windows, version 29.0 (IBM Corp., Armonk, NY, USA), with significance set at *p* < 0.05. Continuous variables were assessed for normality using the Shapiro–Wilk test. Differences in continuous clinical parameters between patients with and without adverse pathology were evaluated using the Mann–Whitney U test, while categorical variables were compared using the Chi-square test. To identify independent preoperative predictors of adverse pathology while avoiding collinearity between overlapping biopsy thresholds, separate multivariable logistic regression models were constructed for the 10% and 15% pattern 4 cutoffs, adjusting for significant clinical covariates. Model discrimination was evaluated using the area under the receiver operating characteristic curve (AUC), and calibration was assessed via the Hosmer–Lemeshow test.

## 3. Results

### 3.1. Patient Selection and Baseline Characteristics

A total of 127 patients with biopsy-confirmed ISUP GG 2 prostate cancer were included. All included patients had a confirmed histopathological diagnosis of prostatic adenocarcinoma, with the explicit absence of cribriform architecture or intraductal carcinoma components on the diagnostic biopsy. Median age was 63.2 years (42–77), median PSA 7.6 ng/mL (2.88–27.33), and median PSA density (PSAD) 0.18 ng/mL/cc (0.05–1). PI-RADS ≥ 4 lesions were present in 71.6% of cases, and clinical stage ≥cT3a was recorded in 22.8%. The median biopsy pattern 4 proportion was 21.1% (range 3–45%). Stratified by volume, the distribution of pattern 4 was 9.8% for <10%, 34.4% for 10–14%, 1.6% for 15–19%, 21.3% for 20–24%, and 32.7% for ≥25%. Clinical, demographic and biopsy results are summarized in Table 1.

### 3.2. Pathologic Outcomes

At final pathology, upgrading to ISUP GG ≥ 3 occurred in 27 patients (21.3%) and upstaging to ≥pT3 in 22 (17.3%), resulting in an overall adverse pathology (AP) rate of 30.7% (39/127). Pathological features of the radical prostatectomy specimens are detailed in Table 2. No pathological downgrading to ISUP GG 1 events was observed. Extraprostatic extension was present in 12.6% (16/127), seminal vesicle invasion in 4.7% (6/127), and lymph-node invasion in 1.6% (2/127) of patients who underwent pelvic lymph node dissection. Positive surgical margins (PSMs) were found in 7.1% (9/127), with no differences in PSM between groups according to AP (*p* > 0.05). Adverse final pathology was observed in 25.0% of men with biopsy pattern 4 < 10% vs. 29.1% with ≥10%, and in 20.4% with biopsy pattern 4 < 15% vs. 35.3% ≥ 15%.

### 3.3. Oncologic Outcomes

After a mean follow-up of 32.3 months (2–109), biochemical recurrence (BCR) was documented in 23 patients (18.1%), with a median time to recurrence of 18.1 months (2–53.9). The 3-year recurrence-free survival (RFS) rate was 81.2% (95% CI: 73.6–88.8%), with 48 patients remaining at risk at the 36-month landmark. On univariable Cox proportional hazards analysis, neither maximum core pattern 4 extent (OR = 0.997, 95% CI: 0.967–1.1027, *p* = 0.832) nor PSAD (OR = 4.76, 95% CI: 0.38–60.19, *p* = 0.228) was significantly associated with the risk of biochemical recurrence.

All 23 patients with BCR underwent PSMA PET/CT restaging. Overall, 15 patients (65.2%) demonstrated a negative PSMA PET/CT with no evidence of disease. Among those with positive scans, local relapse in the prostate bed was identified in three patients (8.7%), regional nodal relapse in four patients (17.4%), and solitary bone metastasis in one patient (4.3%).

In univariable Cox proportional hazards analysis, no preoperative clinical or radiological variables significantly predicted biochemical recurrence. Findings from restaging PSMA PET/CT scans were analyzed purely descriptively due to the limited number of positive scan events (n = 8).

On univariable analysis, preoperative PSA (*p* = 0.028), PSAD (*p* = 0.011), and biopsy pattern 4 proportion ≥ 10% (*p* = 0.042) and ≥15% (*p* = 0.027) were significantly associated with AP (Table 3). MRI features suggesting ≥cT3a stage and PI-RADS ≥ 4 lesions showed a trend toward significance (*p* = 0.081 and *p* = 0.093, respectively). In multivariable logistic regression, PSAD remained an independent predictor of adverse pathology (*p* = 0.005, OR = 6.75, 95% CI: 3.51–12.87). In separate multivariable models, biopsy pattern 4 proportion ≥ 10% (*p* = 0.036, OR = 2.54, 95% CI: 1.15–6.54) and ≥15% (*p* = 0.023, OR = 2.74, 95% CI: 1.06–6.06) were independently associated with adverse pathology (Table 4).

The model’s discrimination for adverse pathology was good, with an area under the receiver operating characteristic curve (AUC) of 0.78 (95% CI 0.71–0.85), and calibration by the Hosmer–Lemeshow test was satisfactory (*p* = 0.42).

Neither clinical nor radiological preoperative variables predicted BCR or PSMA PET/CT positivity at follow-up. Overall, the 3-year recurrence-free survival (RFS) rate was 81.3% for the entire cohort. Exploratory analysis revealed that patients with AP had a shorter median recurrence-free survival compared to those without AP (28 vs. 42 months, log-rank *p* = 0.049), although the difference lost significance in multivariable Cox regression (*p* = 0.11).

Functional outcomes were available for 118 patients (92.9%): at 12 months, continence (defined as 0–1 pad/day) was achieved in 89.0%, and potency recovery (IIEF-5 ≥ 17 or successful intercourse with/without PDE5 inhibitors) in 61.4% of preoperatively potent men. Functional recovery did not differ between patients with or without AP.

## 4. Discussion

In this single-institution cohort of 127 men with biopsy ISUP Grade Group (GG) 2 prostate cancer undergoing RARP, almost one-third harbored adverse pathology (AP, defined as pT3 and/or upgrading to ≥GG3) at final histology despite favorable baseline characteristics and contemporary staging. A higher proportion of Gleason pattern 4 on biopsy and elevated PSA density (PSAD) emerged as independent predictors of AP, whereas traditional clinical or MRI variables did not retain significance in multivariable analysis. Neither preoperative parameters nor AP alone reliably predicted biochemical recurrence (BCR) or PSMA PET/CT-positive disease during follow-up, although AP was associated with shorter recurrence-free survival on univariable analysis [1].

The EAU–EANM–ESTRO–ESUR–ISUP–SIOG guidelines recognize active surveillance (AS) as the standard management for low-risk prostate cancer and as an option for selected patients with “favorable” intermediate-risk disease, typically GG2 with PSA < 10 ng/mL, ≤cT2a, limited tumor volume, and a low pattern 4 component [17,18,19]. However, the strength of evidence for AS in unselected GG2 is lower than for GG1, and guideline panels emphasize the higher risk of progression and metastasis in intermediate-risk patients [17,19,20]. Large AS cohorts and pooled analyses, including the GAP3 initiative and long-term institutional series, consistently report excellent 10–15-year metastasis-free and cancer-specific survival in low-risk disease [3,4,5,18], but higher treatment conversion and progression rates among GG2 men even under protocolled surveillance [3,4,6]. Within this context, the present study provides quantitative thresholds for pattern 4 burden and PSAD that help operationalize the “favorable GG2” concept advocated by contemporary guidelines.

Recent evidence indicates that, in carefully monitored men with ISUP GG2 prostate cancer, delaying radical prostatectomy after upgrading during active surveillance does not compromise oncological outcomes, with recurrence risk driven mainly by PSA and tumor burden rather than timing of surgery [7].

Several pathological and clinical series have highlighted the prognostic importance of Gleason pattern 4 burden within GG2 disease [9,10,11]. Percent pattern 4 has been shown to correlate with adverse pathology, BCR, and metastasis-free survival, and to refine risk stratification beyond binary GG2 labeling alone [9,10]. For example, increasing percent pattern 4 within Gleason 3+4 cancers has been associated with stepwise deterioration in oncologic outcomes, including higher rates of adverse pathology and BCR after radical prostatectomy [11]. Similarly, AS and RP series suggest that men with GG2 and very low pattern 4 burden (<10–15%) have metastasis-free survival approximating that of GG1 cohorts, whereas those with higher pattern 4 involvement show a steeper decline beyond 10–15 years of follow-up [3,4,9,10]. In the current cohort, biopsy pattern 4 thresholds of 10% and 15% were independently associated with 2.5- to 3-fold higher odds of AP after adjustment for PSA and other covariates and contributed to a predictive model with good discrimination (AUC 0.78). These findings reinforce the clinical relevance of explicitly quantifying pattern 4 burden in biopsy reports and support the incorporation of numeric cutoffs into AS eligibility criteria and counseling for GG2 patients [9,10,18].

This study suggests that a pattern 4 cutoff of <10% identifies patients with a lower probability of adverse pathology, which may help refine candidate selection for active surveillance. Furthermore, our findings suggest that extending consideration up to 15% pattern 4 may be feasible in carefully selected patients, although this approach should be individualized given the increasing risk of adverse pathology (OR 2.74, 95% CI 1.06–6.06) [10,12].

PSAD is now recognized as a key component of prostate cancer risk calculators and AS decision tools and is specifically emphasized in the EAU guidelines for biopsy indication and AS selection [16,18]. A PSAD threshold around 0.15–0.20 ng/mL/cc is repeatedly associated with lower risk of clinically significant cancer and with more indolent disease during AS [4,12,18]. In multiple AS cohorts and prediction models (e.g., Johns Hopkins, PRIAS, Canary PASS), low PSAD has emerged as one of the strongest predictors of the absence of upgrading or adverse pathology at repeat biopsy or subsequent radical treatment [3,4,18,20]. In the present analysis, PSAD remained an independent predictor of AP with an odds ratio of approximately 6.7, and in combination with pattern 4 burden it allowed clinically meaningful stratification of pathological risk. MRI variables (PI-RADS 4–5, suspected cT3a) demonstrated only trends towards significance, which is concordant with evidence that mpMRI improves initial selection and reduces undersampling but cannot fully substitute for quantitative histological and PSA-based metrics in predicting adverse pathology [12,13]. Integrating biopsy pattern 4 percentage and PSAD with MRI findings and established AS calculators may therefore provide the most robust preoperative risk assessment in GG2 disease [12,16,18].

Modern reviews caution that AS in unselected GG2 patients carries higher risks of progression and metastasis than AS in GG1 and should not be routine for all intermediate-risk men [18,19,20]. Nonetheless, multiple series and meta-analyses indicate that within intermediate-risk disease there exists a subset of “favorable GG2” patients—typically PSA <10 ng/mL, ≤cT2a, low tumor volume, absence of cribriform or intraductal carcinoma, and minimal pattern 4 burden—whose long-term outcomes on AS are comparable to those of low-risk cohorts [3,4,5,6,17,18]. Active surveillance can be safely considered for carefully selected intermediate-risk patients with limited pattern 4 and favorable clinical features, with 10–15-year metastasis-free and cancer-specific survival still exceeding 90% in published cohorts [18]. The current data support this paradigm by demonstrating that biopsy pattern 4 burden and PSAD discriminate between GG2 patients with a high probability of organ-confined, non-upgraded pathology and those at markedly increased risk for AP at RARP [9,10,12]. In clinical practice, men with GG2 disease, PSAD below commonly used thresholds, and biopsy pattern 4 involvement below 10–15%, particularly in the absence of adverse MRI or histologic features (cribriform/intraductal carcinoma), appear to be the most plausible candidates for AS within GG2, although prospective validation in dedicated surveillance cohorts is required [16,17,21,22].

Despite a relatively high rate of AP, mid-term oncologic outcomes in this series were acceptable, with an 18.1% BCR rate at a mean follow-up of 32 months and low rates of PSMA PET/CT-documented metastatic disease, findings that are broadly consistent with other GG2 cohorts treated with immediate surgery [6,8,14]. The inability of preoperative variables and AP to robustly predict BCR in multivariable models likely reflects limited follow-up duration, modest event numbers, and the biological heterogeneity within GG2, a limitation shared by other GG2 studies and AS cohorts [3,4,6]. Additional limitations of the present work include its single-center, retrospective design, the potential selection bias towards surgically managed patients (excluding those already selected for AS), and the absence of an AS comparison arm, which precludes direct assessment of surveillance safety in this population [6,17,20]. Nevertheless, by focusing on a homogeneous biopsy GG2 cohort worked up with contemporary mpMRI and PSMA PET/CT when indicated, this study offers clinically relevant, easy-to-apply thresholds for pattern 4 burden and PSAD that can be directly tested in future AS protocols and prospective decision tools [9,10,12,17,21].

Finally, the quantification of biopsy Gleason pattern 4 burden relies on visual estimation by pathologists, which introduces inherent inter- and intra-observer variability. Although our statistical models identified precise percentage thresholds (<10% and up to 15%), these exact cutoffs should be interpreted in the context of the visual binning and rounding methods inherent to pathological assessment. Future studies employing digital pathology and automated image analysis algorithmically quantifying pattern 4 surface area may be necessary to validate these exact numerical thresholds with greater objective reproducibility. 

Additionally, minor technical evolution occurred over the study period (2016–2024), including the institutional transition from transrectal to transperineal biopsy approaches and updates in MRI hardware/sequences. Although all historical MRI scans were retrospectively standardized to PI-RADS v2.1 and fusion-targeting software was consistently utilized, potential subtle variability stemming from longitudinal updates in imaging technology and the biopsy access route cannot be completely excluded.

Future research should prospectively validate pattern 4 and PSAD thresholds in GG2-specific AS cohorts, ideally within large collaborative infrastructures such as GAP3, and examine their integration with MRI-based progression criteria and genomic risk classifiers [17,18,23,24,25,26]. Dynamic, risk-adapted surveillance schedules that combine serial PSA, PSAD, MRI changes, and biopsy pattern 4 evolution may reduce unnecessary biopsies and overtreatment while preserving oncologic safety in carefully selected favorable GG2 patients [16,17,21,22,27,28,29,30]. Ultimately, the goal is a personalized management strategy in which GG2 disease is decomposed into biologically and prognostically distinct subgroups and treatment—immediate radical therapy versus AS—is guided by integrated clinical, imaging, and pathological risk, rather than by Gleason grade group alone [17,18,20,22].

## 5. Conclusions

In this contemporary cohort of men with biopsy ISUP Grade Group 2 prostate cancer, PSA density and biopsy Gleason pattern 4 proportion (<10% and <15%) were identified as the strongest independent preoperative predictors of organ-confined, non-upgraded pathology after radical prostatectomy. Combining a low pattern 4 burden with a low PSA density helps operationalize the concept of “favorable intermediate-risk” disease in clinical practice. These quantitative thresholds provide objective tools for patient counseling and may refine eligibility criteria for active surveillance protocols, minimizing overtreatment without compromising oncologic safety.

## Figures and Tables

**Table 1:** Baseline preoperative characteristics: biopsy-confirmed ISUP 2 with all data available (n = 127).

Variable	Value
Age, median (years)	63.2
PSA, median (range), ng/mL	7.6 (2.88–27.33)
PSA density, median (range)	0.18 (0.05–1.0)
PI-RADS ≥ 4, n (%)	91 (71.6)
Clinical stage ≥ cT3, n (%) *	29 (22.8)
Number of positive cores, median (range)	4 (1–14)
Pattern 4 proportion on biopsy, median % (range)	21.1 (3–45)

* MRI staging.

**Table 2:** Final pathological outcomes after RARP.

N (%)/Value	Outcome
27 (21.3)	ISUP upgrading to ≥ 3
0 (0)	ISUP downgrading
22 (17.3)	Pathological stage ≥ pT3
2 (1.6)	Lymph-node invasion (pN1)
9 (7.1%)	Surgical margins (R1)
52.62 (16.60–140.91)	Prostate weight, mean (range), g

**Table 3:** Univariate logistic regression for adverse pathology (≥pT3 and/or upgrading to ≥ISUP 3).

Variable	*p*-Value
PSA	0.028
PSA density	0.011
Pattern 4 proportion ≥ 10%	0.042
Pattern 4 proportion ≥ 15%	0.027
PI-RADS > 3	NS
Radiological clinical stage ≥ cT3	NS

**Table 4:** Multivariate logistic regression for adverse pathology.

Variable	*p*-Value	95% CI	OR
PSA density	0.005	3.512–12.873	6.754
Pattern 4 proportion ≥ 10%	0.036	1.148–6.539	2.536
Pattern 4 proportion ≥ 15%	0.023	1.061–6.061	2.74

## Data Availability

The data presented in this study are available on request from the corresponding author.
